# Supplementary material for: Electrophysiological Indices of Hierarchical Speech Processing Differentially Reflect the Comprehension of Speech in Noise
Source: eNeuro. 2026 Sep 8;13(9):ENEURO.0069-26.2026. doi: 10.1523/ENEURO.0069-26.2026 (PMC13560466; doi:10.1523/ENEURO.0069-26.2026)
Supplement: Supplementary file 1 [file eneuro-13-ENEURO.0069-26.2026-s002.docx]

**Figure 2-1. Pairwise Wilcoxon rank sum test results for behavioral score comparisons across conditions.**

Wilcoxon rank sum tests (two sided) comparing percentage of words heard (PWH) and comprehension scores across experimental conditions. Reported p-values are Holm-corrected for multiple comparisons. Each cell reflects the statistical comparison between the corresponding pair of conditions.

| **PWH** | | | | | |
| --- | --- | --- | --- | --- | --- |
|  | | Quiet | +3 dB | -3 dB | -6 dB |
| a | +3 dB | 3.180 x 10^-4^ | - | - | - |
|  | -3 dB | 1.294 x 10^-4^ | 1.294 x 10^-4^ | - | - |
|  | -6 dB | 1.294 x 10^-4^ | 1.294 x 10^-4^ | 1.294 x 10^-4^ | - |
|  | -9 dB | 1.294 x 10^-4^ | 1.294 x 10^-4^ | 1.294 x 10^-4^ | 1.294 x 10^-4^ |
| **Comprehension** | | | | | |
|  | | Quiet | +3 dB | -3 dB | -6 dB |
| b | +3 dB | 0.599 | - | - | - |
|  | -3 dB | 1.899 x 10^-4^ | 4.904 x 10^-4^ | - | - |
|  | -6 dB | 1.259 x 10^-4^ | 1.259 x 10^-4^ | 1.411 x 10^-4^ | - |
|  | -9 dB | 1.259 x 10^-4^ | 1.259 x 10^-4^ | 1.259 x 10^-4^ | 2.245 x 10^-4^ |
