# Supplementary material for: Electrophysiological Indices of Hierarchical Speech Processing Differentially Reflect the Comprehension of Speech in Noise
Source: eNeuro. 2026 Sep 8;13(9):ENEURO.0069-26.2026. doi: 10.1523/ENEURO.0069-26.2026 (PMC13560466; doi:10.1523/ENEURO.0069-26.2026)
Supplement: Supplementary file 2 [file eneuro-13-ENEURO.0069-26.2026-s003.docx]

**Figure 4-1.** **Generalized linear mixed-effects model results relating partial correlation coefficients to behavioral scores**

Generalized linear mixed-effects models (GLMMs, Eq. 5) relating partial correlation coefficients to percentage of words heard (PWH) and comprehension scores across signal to noise ratios (SNRs). For each model, the intercept, fixed-effects, standard errors, z-values, and associated p-values are reported. Significance levels are denoted as: *p < 0.05, **p < 0.01, ***p < 0.001.

| **PWH GLMM** | | | | | | | |
| --- | --- | --- | --- | --- | --- | --- | --- |
|  | Fixed Effect | | Estimate | Std. Error | z-value | p-value |  |
|  | Intercept | | 1. 609 | 0.138 | 11.635 | 2.726 x 10^-31^ | *** |
|  | Ons_A_ | | -0.129 | 0.086 | -1.497 | 0.134 |  |
|  | Sgram | | 0.117 | 0.088 | 1.322 | 0.186 |  |
|  | Fea | | 0.104 | 0.102 | 1.022 | 0.307 |  |
| p | Surp | | 0.398 | 0.097 | 4.128 | 3.652 x 10^-5^ | *** |
|  | Ons_W_ | | 0.055 | 0.070 | 0.779 | 0.436 |  |
| o | SNR | | 2.133 | 0.104 | 20.454 | 5.521 x 10^-93^ | *** |
| r | SNR:Ons_A_ | | -0.258 | 0.092 | -2.819 | 4.820 x 10^-3^ | ** |
|  | SNR:Sgram | | 0.038 | 0.073 | 0.528 | 0.597 |  |
|  | SNR:Fea | | 0.098 | 0.111 | 0.877 | 0.381 |  |
| q | SNR:Surp | | 0.265 | 0.098 | 2.694 | 7.067 x 10^-3^ | ** |
|  | SNR:Ons_W_ | | 0.083 | 0.071 | 1.165 | 0.243 |  |
| **Comprehension GLMM** | | | | | | | |
|  | Fixed Effect | Estimate | | Std. Error | z-value | p-value |  |
|  | Intercept | 1.046 | | 0.111 | 9.402 | 5.365 x 10^-21^ | *** |
|  | Ons_A_ | -0.015 | | 0.062 | -0.244 | 0.807 |  |
| t | Sgram | 0.128 | | 0.063 | 2.031 | 0.042 | * |
|  | Fea | 0.055 | | 0.060 | 0.919 | 0.358 |  |
| u | Surp | 0.122 | | 0.061 | 1.996 | 0.046 | * |
|  | Ons_W_ | 0.044 | | 0.051 | 0.871 | 0.384 |  |
| s | SNR | 0.830 | | 0.070 | 11.932 | 8.091 x 10^-33^ | *** |
|  | SNR:Ons_A_ | -0.033 | | 0.055 | -0.598 | 0.550 |  |
|  | SNR:Sgram | 0.051 | | 0.049 | 1.022 | 0.307 |  |
| v | SNR:Fea | -0.124 | | 0.058 | -2.137 | 0.033 | * |
|  | SNR:Surp | -0.024 | | 0.053 | -0.462 | 0.644 |  |
|  | SNR:Ons_W_ | 0.038 | | 0.050 | 0.777 | 0.437 |  |
