# Supplementary material for: Electrophysiological Indices of Hierarchical Speech Processing Differentially Reflect the Comprehension of Speech in Noise
Source: eNeuro. 2026 Sep 8;13(9):ENEURO.0069-26.2026. doi: 10.1523/ENEURO.0069-26.2026 (PMC13560466; doi:10.1523/ENEURO.0069-26.2026)
Supplement: Supplementary file 3 [file eneuro-13-ENEURO.0069-26.2026-s004.docx]

**Table 4-1. Estimated effects of lexical surprisal on word reconstruction accuracy across signal-to-noise ratios**

Estimated slopes describing the relationship between lexical surprisal and word reconstruction accuracy at each signal-to-noise ratio (SNR), derived from the linear mixed-effects model in Table 4. Slopes represent the change in reconstruction accuracy associated with surprisal within each noise condition. Values are reported with standard errors, z-ratios, and associated p-values testing whether each slope differs from zero. Significance levels are denoted as: *p < 0.05, **p < 0.01, ***p < 0.001.

|  | SNR | Surp:SNR Slope | Std. Error | z-ratio | p-value |  |
| --- | --- | --- | --- | --- | --- | --- |
|  | Quiet | 0.029 | 2.709 x 10^-3^ | 10.519 | 3.518 x 10^-25^ | *** |
|  | +3 dB | 0.028 | 2.701 x 10^-3^ | 10.336 | 4.614 x 10^-18^ | *** |
|  | -3 dB | 0.024 | 2.730 x 10^-3^ | 8.787 | 1.903 x 10^-11^ | *** |
| ab | -6 dB | 0.019 | 2.709 x 10^-3^ | 6.814 | 3.923 x 10^-6^ | *** |
| ac | -9 dB | 0.013 | 2.736 x 10^-3^ | 4.615 | 1.930 x 10^-24^ | *** |
