# Supplementary material for: Electrophysiological Indices of Hierarchical Speech Processing Differentially Reflect the Comprehension of Speech in Noise
Source: eNeuro. 2026 Sep 8;13(9):ENEURO.0069-26.2026. doi: 10.1523/ENEURO.0069-26.2026 (PMC13560466; doi:10.1523/ENEURO.0069-26.2026)
Supplement: Supplementary file 4 [file eneuro-13-ENEURO.0069-26.2026-s005.docx]

**Table 4-2. Pairwise comparisons of lexical surprisal effects across signal-to-noise ratios**

Pairwise comparisons between lexical surprisal effects across signal-to-noise ratios (SNRs) based on the slopes reported in Table 4-1. Each contrast tests whether the effect of lexical surprisal on word reconstruction accuracy differs between noise levels. Estimates are reported with standard errors, z-ratios, and adjusted p-values corrected for multiple comparisons. Significance levels are denoted as: *p < 0.05, **p < 0.01, ***p < 0.001.

|  | Contrast | Estimate | Std. Error | z-ratio | p-value |  |
| --- | --- | --- | --- | --- | --- | --- |
| x | Quiet – + 3 dB | 2.172 x 10^-4^ | 3.53 x 10^-3^ | 0.062 | 0.951 |  |
| y | Quiet – -3 dB | 4.767 x 10^-3^ | 3.50 x 10^-3^ | 1.363 | 0.580 |  |
| z | Quiet – -6 dB | 9.900 x 10^-3^ | 3.51 x 10^-3^ | 2.817 | 0.034 | * |
| aa | Quiet – -9 dB | 1.600 x 10^-2^ | 3.50 x 10^-3^ | 4.570 | 4.885 x 10^-5^ | *** |
|  | -3 dB – + 3 dB | -4.459 x 10^-3^ | 3.53 x 10^-3^ | -1.290 | 0.580 |  |
|  | -3 dB – -6 dB | 5.133 x 10^-3^ | 3.52 x 10^-3^ | 1.458 | 0.580 |  |
|  | -3 dB – -9 dB | 1.123 x 10^-2^ | 3.50 x 10^-3^ | 3.211 | 0.011 | * |
|  | -6 dB – + 3 dB | -9.683 x 10^-3^ | 3.53 x 10^-3^ | -2.732 | 0.038 | * |
|  | -6 dB – -9 dB | 6.097 x 10^-3^ | 3.54 x 10^-3^ | 1.729 | 0.419 |  |
|  | -9 dB – + 3 dB | -1.578 x 10^-2^ | 3.53 x 10^-3^ | -4.468 | 7.120 x 10^-5^ | *** |
